# The Magnetic Proximity Effect Induced Large Valley Splitting in 2D InSe/FeI_2_ Heterostructures

**DOI:** 10.3390/nano10091642

**Published:** 2020-08-21

**Authors:** Yifeng Lin, Changcheng Zhang, Lixiu Guan, Zhipeng Sun, Junguang Tao

**Affiliations:** 1 School of Materials Science and Engineering, Hebei University of Technology, Tianjin 300130, China; Hebut8124@sohu.com; 2 School of Science, Hebei University of Technology, Tianjin 300401, China; hebut2017@139.com (C.Z.); hebut2016@126.com (Z.S.)

**Keywords:** InSe, magnetic proximity effect, first-principles calculations, spin-orbit coupling

## Abstract

The manipulation of valley splitting has potential applications in valleytronics, which lacks in pristine two-dimensional (2D) InSe. Here, we demonstrate that valley physics in InSe can be activated via the magnetic proximity effect exerted by ferromagnetic FeI_2_ substrate with spin-orbit coupling. The valley splitting energy can reach 48 meV, corresponding to a magnetic exchange field of ~800 T. The system also presents magnetic anisotropy behavior with its easy magnetization axis tunable from in-plane to out-of-plane by the stacking configurations and biaxial tensile strain. The *d*-orbital-resolved magnetic anisotropic energy contributions indicate that the tensile strain effect arises from the increase of hybridization between minority Fe *d_xy_* and $d_{x^{2}-y^{2}}$ states. Our results reveal that the magnetic proximity effect is an effective approach to stimulate the valley properties in InSe to extend its spintronic applications, which is expected to be feasible in other group-III monochalcogenides.

## 1. Introduction

The contemporary semiconductor industry demands exponentially growing computational power of integrated circuits with continuous decreasing physical size of the transistors to only a few nanometers. This requires new electronic devices with the implementation of spintronic components that use electron spins as information memory, not just its charge properties. Since the discovery of graphene [1,2], two-dimensional (2D) materials have displayed immense potential in ultrathin electronic, spintronic, and optoelectronic devices due to their highly tunable and exotic physical properties. Besides graphene, many other 2D materials have been extensively studied so far, such as transitional-metal dichalcogenides (TMDCs) [3,4,5], black phosphorus (BP) [6,7,8,9], and group-III monochalcogenides (MX, M  =  Ga, In; X  =  S, Se, Te) [7,10,11,12], etc. The rich electrical and optical properties as well as the presence of ferromagnetism in 2D materials create ample opportunities for possible integration of information processing and storage. In addition to charge and spin degrees of freedom, the valley is a new distinctive electron freedom that comes from the degenerate energy extremes at special *k* points in the Brillouin zone (BZ) and involves manipulating and using the valley index as an information carrier. Through spin-orbit coupling (SOC), the spin and valley can be locked, making it possible to link these two basic quantum information. The valleytronics stores information and performs logic operations that complement or surpass conventional semiconductor technologies. Because of the broken inversion symmetry, monolayer TMDCs feature two inequivalent valleys with different angular momenta. However, these two valleys are energetically degenerate and locked by the time-reversal symmetry [9,13]. The spin-valley locking has a long lifetime, because the valleys are well separated in momentum space [13]. However, the carrier densities at two inequivalent valleys are the same as that required by the respective time-reversal symmetry. For efficient use of the valley degree of freedom, this balance must be broken to create, switch, and detect valley polarization for easily distinguishing and manipulating the carriers at different valleys. To this end, the valley degeneracies are often lifted by introducing a nonzero magnetic moment to the materials through the defect doping of magnetic ions or vacancies, applying a magnetic field, or the magnetic proximity effect (MPE), which may enable the valley-specific band engineering and application of these materials in novel magneto-optical and valleytronic devices. The magnetic doping is difficult to control in experiments, but it facilitates the deposit films on magnetic substrate. The proximity-induced exchange interaction, usually considered as a perturbative effect for bulk materials, can fundamentally alter the electronic structure of the 2D materials. Compared to the magnetic doping, utilizing MPE allows us to avoid the introduction of defects and reliably separate the bulk from the surface state effects.

Manipulating exchange interactions in magnetic heterostructures has been proven to be an effective way to engineer highly functional materials. It has been shown that only a tiny valley Zeeman splitting of 0.1–0.2 meV/T can be achieved using an external magnetic field for almost all TMDCs, due to their similar electronic structures and the same orbital moment contributions [14,15]. On the other hand, the exchange valley splitting by MPE can generate fairly large tunable Zeeman splitting, which depends on the exchange interaction strength between the magnetic substrate and TMDCs, the interlayer separation and band alignment [16]. So far, there are intense studies of 2D materials to achieve the valley splitting via proximity exchange effect [17,18,19,20,21,22,23,24,25]. Theoretical work has predicted strong exchange effects and emergent phenomena in the systems integrating 2D materials with magnetic substances. For instance, many investigations have been performed for TMDCs on magnetic substrates such as EuO [17], EuS [18], MnO [20,21], CoO [19], Cr_2_O_3_ [22], and CrI_3_ [23,24,25], as well as other 2D systems: SnO/CrN [26], and 2H–VS_2_/Cr_2_C [27] to study their interfacial magnetic exchange effect. In addition, the in-plane strain, the vertical electric field and the layer separation can be used to further manipulate and modulate valley splitting in some of the above systems [16,21]. The MPE in TMDCs-based systems are facilitated via the *d–d* orbital interaction from the transition metals involving both the substrate and the functional layers. A natural question arises: can one exploit the exchange interaction to tune both the magnitude and sign of valley splitting of a 2D material without valence *d–d* interaction? The answer to this question will help to deepen the understanding of the origin of the MPE.

Recently, InSe has been widely studied [28,29]. It holds great promise for electronics, optoelectronics and catalysts due to its high carrier mobility, sombrero-shape valence band edges, rare *p*-type electronic behaviors, and unusual nature of the electronic interaction, etc. [30,31,32,33]. Due to the weak electron-phonon scattering, InSe-based field effect transistor (FET) exhibits high carrier mobility of 10^3^ cm^2^/V^−1^·s^−1^ at room temperature [28,34], which is in the same magnitude of BP [7] and much higher than that of TMDCs [35]. Photodetectors based on InSe nanosheets exhibit high photoresponsivity and fast response time within a broad spectral range. It presents anisotropic crystalline structure with layers formed by two deformed hexagonal sublayers. Within each layer, it consists of covalently bonded Se-In-In-Se tetra-layer sheets, with the sheets held together by van der Waals (vdW) forces. The anisotropic crystalline structure leads to strong anisotropy of the electronic structure. Unlike TMDCs, there is no transition metal in InSe. The in-plane lattice constant of InSe is 4.07 Å. Among various reported 2D magnetic materials, the in-plane lattice constant of FeI_2_ [36] has the best match with it. The exfoliation energies of FeI_2_ is 0.92 eV/nm^2^, which is comparable to graphene (0.83 eV/nm^2^) [36], suggesting the feasibility to synthesize FeI_2_ monolayer in experiments. In addition, the magnetic anisotropy is a particularly important parameter in vdW crystals because isotropic spin systems cannot order in 2D due to strong thermal fluctuations [37]. The SOC effect in InSe has also been demonstrated to be very important for its electronic structure and optical transition properties [38,39].

In this work, we performed density functional theory (DFT) calculations to explore the valley-splitting related properties in monolayer InSe induced by the MPE via the coupling with FeI_2_ substrate. Our results show that the valley splitting of InSe between *k* and *k*′ valleys can reach ~48 meV, which is equivalent to an external magnetic field of ~800 T. In addition, the magnetic anisotropic behavior of the system has also been investigated. It is found that the magnetic anisotropic energy (MAE) is sensitive to the stacking sequence and biaxial strain, which can flip its easy magnetization axis from in-plane to out-of-plane. Through the *d*-orbital-resolved MAE analysis, the effect of tensile strain mainly arises from the increase of the hybridization between the minority Fe *d_xy_* and $d_{x^{2}-y^{2}}$ states.

## 2. Calculation Details

InSe crystallizes in a base-centered orthorhombic structure with the space group of *R3m* (No. 160) [40]. All calculations are performed using first-principles calculations based on DFT as implemented in the Vienna Ab-initio Simulation Package (VASP, V5.3, Vienna, Austria) [41,42,43]. The pseudopotentials used are generated by the projected-augmented-wave (PAW) method [43]. The valence configurations of In, Se, Fe and I atoms are 5*s*^2^5*p*^1^, 4*s*^2^4*p*^4^, 3d^7^4s^1^, and 5*s*^2^5*p*^5^, respectively. The Kohn-Sham orbitals are expanded in a plane-wave basis with a cutoff energy of 600 eV, and the exchange-correlation functional is treated by Perdew-Burke-Ernzerhof form generalized gradient approximation (GGA-PBE) [44]. The Brillouin zone (BZ) is sampled by 15 × 15 × 1 Monkhorst-Pack *k*-point grids. All atom positions are fully optimized until the Hellman-Feynman forces are smaller than 0.001 eV/Å. The vacuum spacing between neighboring supercells are set to 20 Å to avoid artificial interactions. In addition, the vdW interaction is taken into account using the Grimme’s DFT-D3 method [45]. The SOC effect is considered when necessary.

MAE is defined as the energy difference between the in-plane and out-of-plane magnetization directions, MAE *= E*^[100]^ − *E*^[001]^, where *E*^[100]^ and *E*^[001]^ represent the total energies of the in-plane and out-of-plane magnetization directions. The negative and positive values indicate in-plane magnetic anisotropy (IMA) and perpendicular magnetic anisotropy (PMA). Based on the second-order perturbation theory, the MAE is determined by the spin-orbit matrix element differences following the equation:

(1) $$\mathrm{MAE}\propto \xi^{2}\underset{o,u}{\sum }\frac{\left|<o\right|{\hat{L}}_{z}|u>{|}^{2}-\left|<o\right|{\hat{L}}_{x}|u>{|}^{2}}{E_{u}-E_{0}}$$

where *ξ* is the spin-orbit coupling constant, *|o>* and *|u>* indicate the occupied and unoccupied states, respectively. *E*_o_ and *E*_u_ are the eigen-energies of the occupied and unoccupied states, respectively. And ${\hat{L}}_{x}$ and ${\hat{L}}_{z}$ are angular momentum operators in *x* and *z* directions. The small energy separation (*E*_u_ − *E*_o_) between the occupied and unoccupied states is responsible for the variation in MAE [46]. The *d*-orbital-resolved MAE is determined by the spin-orbit matrix element differences and their difference in energy between in-plane and out-of-plane [27,47].

## 3. Results and Discussion

### 3.1. Electronic Structure

**Figure 1:**
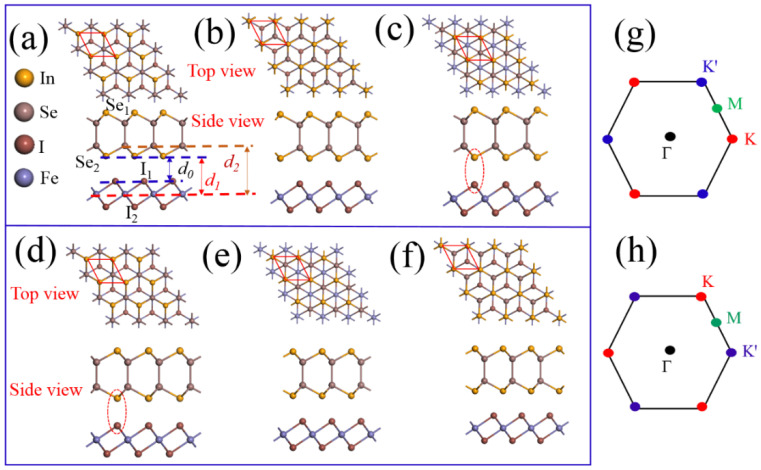
Top and side views of InSe/FeI_2_ heterostructures in six stacking configurations: namely, C-1 (**a**), C-2 (**b**), C-3 (**c**), C-4 (**d**), C-5 (**e**) and C-6 (**f**). The red dotted line indicates the unit cell of the heterostructures. *d*_0_, *d*_1_, and *d*_2_ are the interlayer distance, Fe-Se separation and Fe–In separation, respectively. (**g**,**h**) are the first Brillouin zones for C-1 to C-3 and C4 to C-6, respectively. The high-symmetry point K for C-1 to C-3 corresponds to the K′ point for C-4 to C-6.

Based on the optimized structures, the monolayer InSe and FeI_2_ slabs have in-plane lattice constants of 4.07 Å and 3.98 Å, respectively. With the small lattice mismatch of 2.2%, it is convenient to construct their 1 × 1 heterostructure. We fix the in-plane lattice constant of InSe/FeI_2_ heterostructure to the value of InSe. Thus, a small tensile strain is applied in monolayer FeI_2_. We investigated six possible stacking configurations of the InSe/FeI_2_ heterostructure by considering high symmetrical positions, named C-1 to C-6 as shown in Figure 1a–f, respectively. In C-1, In and Se atoms are on the direct top of Fe and bottom layer I (I2) atoms, respectively, while the top layer I (I1) atoms are located in the hexagonal hollow sites of In-Se rings. In C-2, In and Se atoms are on the direct top of I1 and Fe atoms, respectively, while the I2 atoms are at the hollow sites. In C-3, In and Se atoms are on top of I2 and I1 atoms, respectively, with the Fe atoms in the hollows. In C-4 to C-6, the InSe are rotated by 180°, the atom arrangement information is as follows: in C-4, In and Se atoms sit on top of Fe and I1 atoms and the I2 atoms are at the hollow sites of In-Se rings. In C-5, In and Se atoms overlaps with I1 and I2 atoms while Fe are located in the hollows. For C-6, In and Se atoms are situated directly on top of I2 and Fe atoms while I1 atoms are located in the hexagonal hollow sites. The interfacial distance (*d*_0_) is defined as the separation in *z* direction of I1 atoms and bottom layer Se (Se2) atoms, as shown in Figure 1a. The calculated *d*_0_, the Fe-Se distance (*d*_1_) and Fe-In distance (*d*_2_) for the six configurations after structure relaxation are tabulated in Table 1. The results reveal that the stacking configurations have a great influence on the interfacial distance. The separations in C-3 and C-4 are much larger than that of the others due to the strong repulsive force between Se2 and I1 atoms that are situated in the same vertical line. As shown in Table 1, C-1 is the most stable configuration and has the smallest interfacial distance. In the following, we will mainly discuss the relevant issues based on this configuration. 

In most of the configurations, the interfacial distances are short implying that the FeI_2_ substrate would cause important impacts on the monolayer InSe. However, since the Fe atoms are sandwiched between two I atomic layers, the distance between Fe and In (Se) layers is relative large, ~4.8 Å (~6.1 Å). Therefore, its magnetic influence is mediated by the internal I1 atoms. Indeed, we find that only a small magnetic moment (~0.004 μ_B_) can be induced in In and Se atoms, while I atoms acquire relative large magnetic moment of ~0.17 μ_B_. Thus, the magnetism of InSe induced by the magnetic FeI_2_ substrate is weak and not very sensitive to the stacking configurations. However, the formation of heterostructure breaks the time-reversal symmetry of monolayer InSe and its valley degeneracy can be lifted, which will be addressed later.

**Figure 2:**
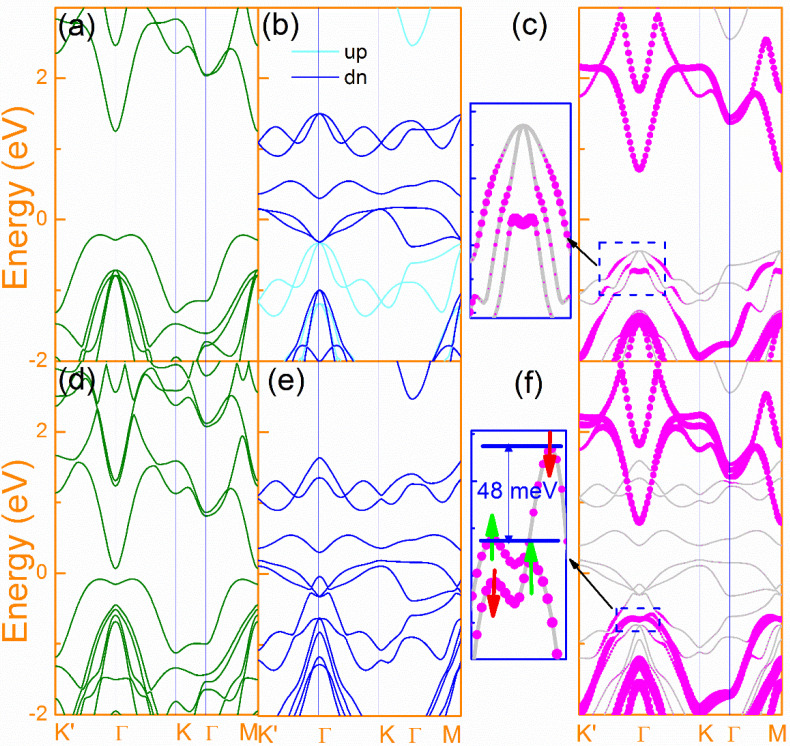
The band structures of InSe (**a**,**d**), FeI_2_ (**b**,**e**) and InSe/FeI_2_ in C-1 configuration (**c**,**f**). The top and bottom panels are the ones without and with SOC. In (**b**), the cyan and blue lines are the spin-up and spin-down components. In (**c**,**f**), the pink solid dots indicate the contributions from InSe with their size reflecting the relative weight. The insets at left part of (**c**,**f**) are the zoom-in views of the valence top of InSe, where the spin and valley splitting in (**f**) are noticed.

In Figure 2, the band structures of InSe, FeI_2_ the InSe/FeI_2_ heterostructure (in C-1 configuration) are compared with and without SOC. The *z* axis is chosen as the quantized direction, and the spin projections for monolayer FeI_2_ along spin-up and spin-down are indicated by the cyan and blue lines in Figure 2b, respectively. From Figure 2a,d, InSe exhibits indirect band gap and its valence-band maximum (VBM) and conduction-band minimum (CBM) reside at the region between the high-symmetry lines along *Γ-K* (*Γ–K*′) and *Γ* points, respectively. This shows that the SOC has strong effect on the band structure of InSe. Surprisingly, the band gap is decreased from 1.46 eV to 0.15 eV due to the downward shift of the conduction band under the effect of SOC. However, there is no band splitting at VBM and the maxima of the sombrero top are degenerated (<2 meV). This sombrero shape band feature is largely originated from the Se *p*_z_ orbitals [48,49]. Since the Se2 is very close to the I1 atom, the interaction between them will have a stronger effect on the band features. On the other hand, the SOC has a minor effect on the band structure of FeI_2_, see Figure 2b,e. When the heterostructure is formed, as can be seen in Figure 2c, the valence band of monolayer InSe is strongly hybridized with the substrate, and its sombrero top characteristic has been partly destroyed. For freestanding monolayer InSe, there is no spin splitting at sombrero top even with SOC, while the spin-up band along *Γ–K* is energy degenerate with the spin-down band along *Γ–K*′ as a result of time-reversal symmetry. However, as shown in Figure 2f, the VBM degeneracy in InSe has been lifted when placed on FeI_2_, which evidences that the MPE-induced valley splitting is robust. Besides, we also calculate the band structures of the other five configurations (see Figure S1 in the Supplementary Information) and find that the valley/spin splitting is a common phenomenon for all stacking configurations. The asymmetric band structure suggests that the spin polarization can be induced in InSe from the interfacial MPE by FeI_2_. In all configurations, InSe strongly hybridizes with FeI_2_, inducing a half-semiconducting character of InSe. By comparing Figure 2c,f, we can find that the MPE of FeI_2_ is significant when SOC is considered. Under the joint action of MPE and SOC, the energy band increases along the direction of *Γ–K*, but decreases along *Γ–K*′, leading to a valley splitting of ~48 meV at VBM. This valley splitting is considerable large as compared to MoS_2_ in MoS_2_/EuS (37.3 meV) and MoS_2_/Cr_2_O_3_ (23.4 meV) heterostructures [18,22]. It should be noted that no *d* electrons are involved in InSe. Thus, the magnetic exchange mechanism in the current system is different from that of TMDCs.

Since the InSe in C-4 to C-6 configurations are rotated 180° around the *z* axis as compared to C-1 to C-3 configurations, the *K* and *K*′ of the Brillouin zone for InSe in C-4 to C-6 configurations are opposite to those in C-1 to C-3 configurations, see Figure 1g,h. However, in Figure 2 and Figure S1, we see that the valley splitting directions of all configurations are the same. In our calculations, the crystal structures are fixed to the symmetry of FeI_2_, which are invariable. Thus, the invariance of valley splitting shows that the valley splitting is determined by FeI_2_. At the same time, even in C-3 and C-4 configurations, the symmetry is broken. However, there is almost no valley splitting for C-3 and C-4, which evidences that proximity magnetic interactions play major roles in such effect than the breaking of symmetry.

**Figure 3:**
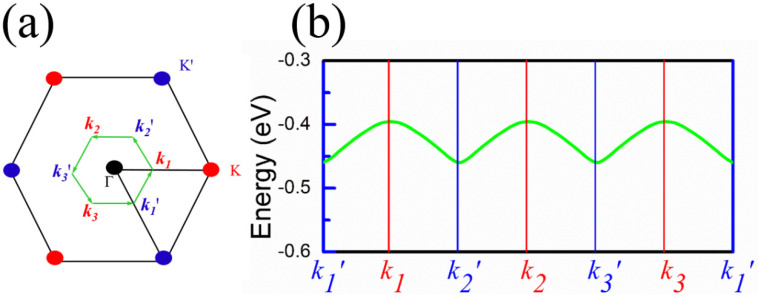
The first Brillouin zones for C-1 configuration (**a**). The green arrows indicate a *k*-point loop from *k*_1_*′* to *k*_3_, where *k_n_* (*n* = 1, 2, 3) (red) and *k_n_′* (*n* = 1, 2, 3) (blue) are the *k*-point positions for the highest band energies of InSe along *Γ–K*, and *Γ–K*′, respectively. (**b**) The top valence band of InSe along the *k*_1_*′* to *k*_3_ *k*-point loop.

Next, we will give solid evidence for the term “*valley*” used above. The band dispersion of freestanding InSe forms a caldera shape for VBM, where the caldera rim is of equal height due to the energy degeneracy along *Γ–K* and *Γ–K*′. However, when the heterostructure is formed, the proximity magnetic field breaks the symmetry, which results in different band dispersions along *Γ–K* and *Γ–K*′. As such, the energy counter of VBM will exhibit valley behavior similar to that of TMDC materials. To illustrate this more clearly, the top band dispersion of InSe is plotted along a closed *k*-point loop of *k*_1_*′ − k*_1_ *− k*_2_*′ − k*_2_ *− k*_3_*′ − k*_3_ *− k*_1_*′*, where *k_n_* (*n* = 1, 2, 3) and *k_n_′* (*n* = 1, 2, 3) are the *k*-point positions in the first Brillion zone where the highest band energies for InSe are located along *Γ–K*, and *Γ–K*′, respectively, see Figure 3a for details. In Figure 3b, it clearly shows that the band energy along this closed loop periodically changes from the high (at *k_n_* points) to low (at *k_n_′* points) values. With this energy variation between *k_n_* and *k_n_′*, the *valley*-like band dispersions will be formed for the VBM of InSe similar to the *K* and *K′* valleys in TMDCs [13]. Clearly, the appearance of these *valley* features should be attributed to the MPE of FeI_2_. As shown in Figure 2 and Figure S1, the valley splitting is robust for most of the configurations, except for C-3 and C-4 configurations where the layer separations are large. In addition, the CBM of all configurations move away from VBM of InSe, leading to larger band-gaps for InSe in contrast to the freestanding one. 

**Figure 4:**
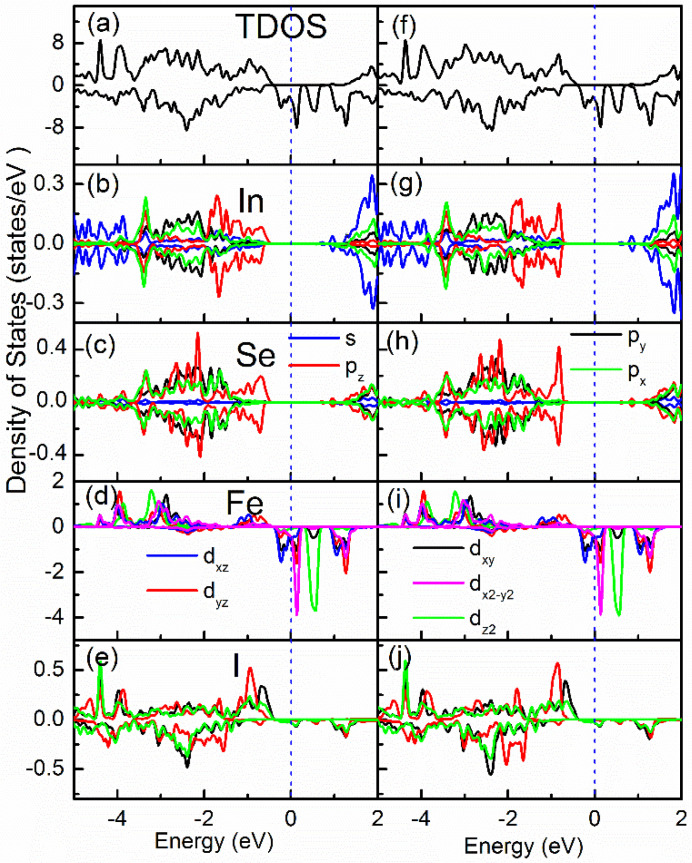
The density of states (DOS) spectra for C-1 (**a**–**e**) and C-3 (**f**–**j**) configurations. The (**a**,**f**) are the total DOS. In (**b**–**e**) and (**g**–**j**), different orbitals are presented by different colors provided in the figure. The vertical dashed blue lines indicate the positions of the E_F_.

To reveal the contributions of different atomic states at the band edges, the total density of states (TDOS) and orbital-resolved partial density of states (PDOS) of InSe/FeI_2_ are compared in Figure 4 for C-1 and C-3. It shows that the spin-up and spin-down states are well separated, and the Fe spin-down orbitals have negligible hybridization with InSe in valence bands. The VBM of InSe are mainly constructed by the In and Se *p_z_* orbitals, which agrees very well with previous reports [48,49]. From Figure 4, we see that tha I atom is antiferromagnetically coupled with the Fe atom. Thus, the spin-down orbitals are lower in energy than the spin-up ones. The antiferromagnetic interaction between Fe and I implies that the magnetic exchange mechanism of FeI_2_ can be super-exchange. Furthermore, the Fe–I–Fe bond angle is very close to 90° (91.6°). Therefore, the ferromagnetic super-exchange is allowed based on Goodenough-Kanamori rules [50]. Comparing C-1 and C-3 configurations, it is found that In and Se atoms in C-1 have relatively large spin asymmetry at the VBM. For example, in Figure 4b,c, the spin-down states for both In and Se atoms are a bit lower in energy than their corresponding spin-up states due to the hybridization with the I atom. This behavior can be almost ignored in C-3. Due to the lack of band splitting, the van Hove singularity in C-3 is more pronounced. There is strong hybridization between the In *p_x_*, *p_y_* and Se *p_x_*, *p_y_* orbitals, which are located at ~1.5 eV away for the VBM of InSe. At the VBM of InSe, there are purely *p_z_* orbitals. Therefore, it is concluded that the In *p_z_* and Se *p_z_* states around the *k_n_* and *k_n_′* points play a key role in the valley splitting. 

In all configurations, the In and Se atoms have hardly any hybridization with Fe. Therefore, the spin properties of InSe are correlated with I, which is indirectly connected with Fe. In Figure S2 (see the Supplementary Information), the spin density distributions of InSe in C-1 and C-3 configurations are compared. Clearly, the spin polarized electrons are more populated around Se2 atoms that are closer to I1 atoms. For C-1 with shorter separation, the spin distribution can be extended to the Se1 atoms, but not on any of the In atoms, although the Se1 atoms are farther from the substrate than the In atoms. This proves that the magnetic interaction with the substrate is conducted by the Se atoms. As shown in Figure S2b, there is no spin distribution on the Se1 atoms in C-3 but there is in C-1, showing that the MPE has a short-range effect. The MPE causes significant variations in the band structure of the InSe/FeI_2_ heterojunction via strong SOC interactions.

From the band structure and density of states (DOS) spectra shown above, we can see that there are only spin down electrons at the Fermi level (E_F_) of the heterostructure, which are mainly from Fe. A 100% spin polarization is formed, which makes the system half-metallic. The half-metallic 2D heterostructures can be used for electrical injections of specific spin into InSe by electric control, which is beneficial for its spintronics applications.

The mechanisms underlying the valley splitting of InSe/FeI_2_ is different from TMDCs systems where the magnetic moments are introduced to the transition metals in the later cases [17]. In TMDCs, the large spin splitting at the top valence bands of *K* and *K*′ points are mainly facilitated by the Dresselhaus SOC effect via the *d* orbitals of transition metals. The Dresselhaus SOC effect and mirror symmetry of monolayer TMDCs make only out-of-plane spin splitting possible. When the symmetry is broken in vdW heterostructures, the Rashba-type SOC effect will be involved. For the free-standing InSe layer, the electrical dipole moments with threefold in-plane rotational symmetry induces Dresselhaus SOC, which can be expressed as [51]:

(2) $$H_{D}=\gamma \left(3k_{x}^{2}-k_{y}^{2}\right)k_{y}\sigma_{z}$$

where *γ* is a coefficient and $\sigma_{z}$ is the Pauli matric. The effective Dresselhaus SOC field is out-of-plane with threefold rotational symmetry. When the inversion symmetry is broken by a substrate, the in-plane Rashba SOC field with isotropic strength will be generated: [51]

(3) $$H_{R}=\alpha \left(k_{x}\sigma_{y}-k_{y}\sigma_{x}\right)$$

where *α* is the Rashba SOC strength, $\sigma_{x\left(y\right)}$ are the Pauli matrices. The Dresselhaus SOC effect already exists for the free-standing InSe. The MPE will mainly tune the strength of Rashba SOC, which exhibits more in-plane contributions. Although the valley-spin locking has been well demonstrated for TMDCs [13], the valley physics of InSe has not been widely discussed before. However, a weak valley-dependent *g* factor in the six shallow VBM on the caldera rim has been reported in previous work [52], which indicates an implicit relationship between the valley and spin. This relationship is strongly coupled through inter-valley scattering, which makes the valley Zeeman effect more difficult to detect. However, as demonstrated here, with the assistant of MPE, the inter-valley scattering strength can be increased, which leads to strong Zeeman valley splitting. In C-1 configuration, the valley splitting is ~48 meV. Since one Bohr magneton is equal to 5.78 × 10^−5^ eV T^−1^, [53] the equivalent magnetic field is in the order of ~800 T for this system. 

### 3.2. Interfacial Charge Transfer

According to the Bader charge analysis, there is a small amount of electron transfer (approximately 0.02 e^−^) from the FeI_2_ to InSe layer, indicating that the interlayer interaction between FeI_2_ and InSe should be vdW force due to so few transferred charges. The charge transfer direction agrees with our work function calculations that the E_F_ of FeI_2_ is about 0.74 eV higher than that of InSe. We note that the charge transfer in these vdW heterostructures is a kind of spatial transfer rather than a chemical-bond transfer, and so the induced magnetic moments in In and Se atoms are very small, as given in Table 1. Nevertheless, the valley degeneracy is lifted by the breaking of time reversal symmetry by the magnetic substrate.

**Figure 5:**
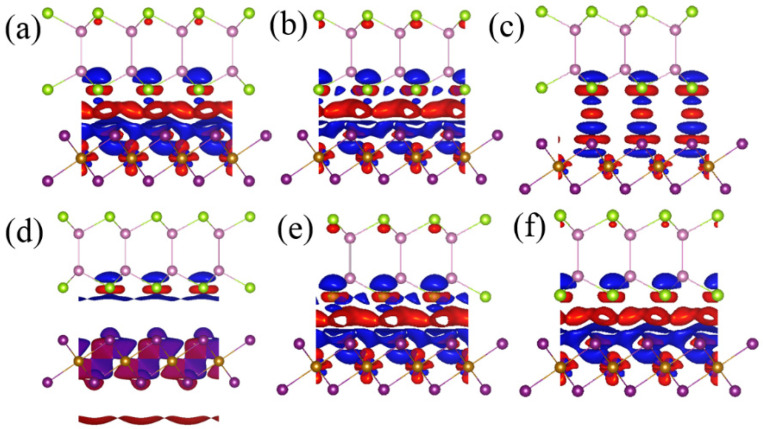
Differential charge density of C-1 to C-6, (**a**–**f**). The red and blue colors represent charge accumulation and depletion, respectively. The isosurface value is of 0.0003 e·Å^−3^. The green, pink, purple and brown balls represent Se, In, I, and Fe atoms, respectively.

Considering that the strength of the MPE is highly dependent on the band hybridization at the interface, the charge density difference (CDD) (∆ρ) distributions for all InSe/FeI_2_ heterostructures are given in Figure 5, which is obtained by:

(4) $$∆\rho =\rho_{hs}-\rho_{InSe}-\rho_{FeI_{2}}$$

where $\rho_{hs}\;$ is the total charge density of the InSe/FeI_2_ heterostructures, and ${\;\rho }_{InSe}$ and $\rho_{FeI_{2}}$ are the charge density of isolated InSe and FeI_2_, respectively. The red regions in Figure 5 stand for electron accumulation and the blue regions stand for electron depletion. Although there is negligible charge transfer from FeI_2_ to InSe, the charges are redistributed in the interfacial region. There is more electron accumulation at the InSe/FeI_2_ interface for C-1, C-2, C-5, and C-6 than C-3 and C-4, indicating the strong band hybridization and electron transfer between FeI_2_ and InSe, which is in consistent with the previous reports on the MoTe_2_/EuO system [17]. From Figure 5a,b,e,f, we can see that the interfacial electrons are more distributed on the InSe side and the electron dissipation happens at the FeI_2_ side. While in InSe, the extra electrons are mostly located in Se2 *p_z_* orbitals. Surprisingly, there are some electron accumulations at the top layer Se1 for C-1, C-2, C-5, and C-6. This indicates that the Se atoms have stronger interactions with the substrate. Since the VBM of InSe is mainly constructed by the Se *p_z_* orbitals, it is then not surprising that it exhibits valley splitting. As compared to C-3 and C-4, the interfacial electron accumulations in C-1, C-2, C-5, and C-6 are delocalized, which suggests its long-range effect. The C-3 and C-4 configurations have negligible valley splitting and their charge accumulations are only localized on some specific atoms. As evidenced above, the C-1, C-2, C-5, and C-6 configurations have stronger valley splitting. Therefore, one can conclude that the charge interfacial electron accumulations play a significant role in their interactions.

### 3.3. Magnetic Anisotropic Properties

**Figure 6:**
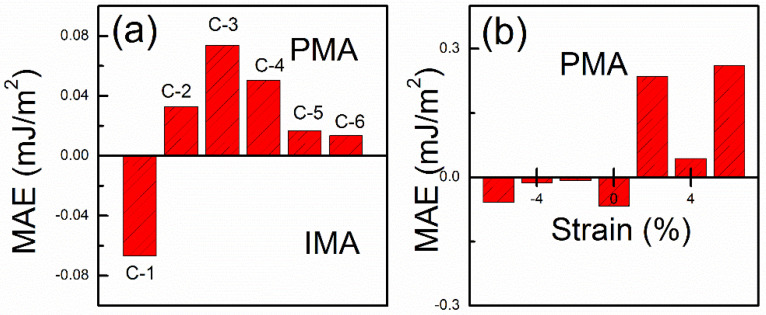
The total magnetic anisotropic energy (MAE) of different configurations (**a**) and C-1 with different strains (**b**). The positive value favors perpendicular magnetic anisotropy (PMA) and negative favor in-plane magnetic anisotropy (IMA). In (**b**), the negative strains stand for the compressive strain, while the positive values represent the tensile strains.

For practical spintronic applications, the magnetic anisotropic effect is also crucial for 2D ferromagnetic materials [27,54]. The easy magnetization axis of monolayer FeI_2_ lies in the *x–y* plane in the previous report [36]. As discussed above, the MPE will mainly tune the in-plane Rashba SOC strength. As a result, the Dresselhaus and Rashba SOC induced perpendicular and in-plane magnetic field will change their relative weight. As such, the MPE induced MAE is expected. To investigate the MAE of 2D InSe/FeI_2_ hetersostructures, we first calculated the total MAE of Fe atom for all configurations, see Figure 6a. Clearly, different stacking configurations affect the MAE of the system. The MAE are −0.067 mJ/m^2^ for C-1 configuration corresponding to −0.069 meV/Fe, which agrees very well with previous study [36]. The negative value for C-1 indicates IMA, which is also in line with previous finding for FeI_2_ [36]. In Table 1, it shows that C-1 is the most stable configuration, and thus its MAE behavior is similar to free-standing FeI_2_. Interestingly, MAE of FeI_2_ for all other five configurations show positive values, i.e., PMA behavior. 

In 2D materials, applying a strain is an important tool to tune the electronic structure. The MPE-induced valley splitting is mediated with the interfacial orbital hybridization between monolayer InSe and FeI_2_. Thus, we expect its magnitude can be modulated by changing the hybridization strength. We applied biaxial strain to the C-1 InSe/FeI_2_ heterostructure to investigate this effect. The range of biaxial strain is −6% to 6%, which is calculated by:

(5) $$\varepsilon =\left(a-a_{0}\right)/a_{0}\times 100\%$$

where *a* and *a*_0_ are the lattice parameters of the strained and free InSe/FeI_2_ heterostructure, respectively. Based on the definition of *ε*, the negative values indicate compressive strain while positive values present tensile strains. In Figure 6b, it is clear that the MAE of the system are all IMA for the compressive strains and change to PMA for the tensile strains. This suggests that the magnetic anisotropy of the system can be easily controlled by external strains to realize the transformation from IMA to PMA.

Although the full relativistic Hamiltonian (including SOC) is based on the total angular momentum eigenstates, and the |*l*,*m_l_*,*m_s_*> is no longer a good quantum number, the MAE can still be obtained with good accuracy by projecting the local density of state into different orbitals and spins [46]. This allows one to investigate the *d*-orbital-resolved MAE to understand the origin of the difference in MAE. Based on the second-order perturbation theory proposed by Wang et al. [46], the orbital-resolved MAE can be obtained by calculating the following two terms:

(6) $$∆E^{--}\propto \xi^{2}\underset{o^{-},u^{-}}{\sum }\frac{\left|<o^{-}\right|{\hat{L}}_{z}|u^{-}>{|}^{2}-\left|<o^{-}\right|{\hat{L}}_{x}|u^{-}>{|}^{2}}{E_{u}^{-}-E_{o}^{-}}$$

(7) $$∆E^{+-}\propto \xi^{2}\underset{o^{+},u^{-}}{\sum }\frac{\left|<o^{+}\right|{\hat{L}}_{z}|u^{-}>{|}^{2}-\left|<o^{+}\right|{\hat{L}}_{x}|u^{-}>{|}^{2}}{E_{u}^{-}-E_{o}^{+}}$$

where + and − are majority and minority spin states, respectively. The difference of the square of the orbital angular momentum matrix elements between the two magnetization directions in Equations (6) and (7), i.e., $\left|<o^{-}\right|{\hat{L}}_{z}{\left|u^{-}>\right|}^{2}-\left|<o^{-}\right|{\hat{L}}_{x}{\left|u^{-}>\right|}^{2}$ and $\left|<o^{+}\right|{\hat{L}}_{z}{\left|u^{-}>\right|}^{2}-\left|<o^{+}\right|{\hat{L}}_{x}{\left|u^{-}>\right|}^{2}$ are given in Table 2.

**Figure 7:**
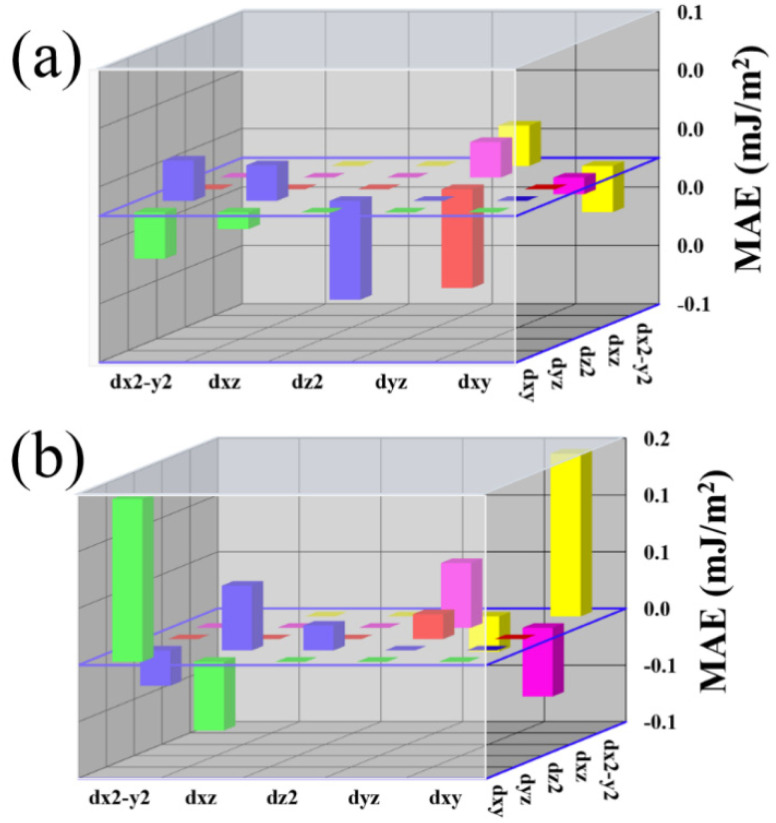
The *d*-orbital-resolved MAE of Fe atom in −6% (**a**), and +6% (**b**) strains in C-1 stacking configuration.

In order to analyze the mechanism of this transformation, *d*-orbital-resolved MAEs under the 6% compressive and tensile strain are compared in Figure 7. At the 6% compressive strain, the main MAE contributions of Fe can be ascribed to the matrix element differences between the (*d_yz_*, $d_{z^{2}}$) orbitals, which is negative. Besides this, the contributions by (*d_xy_*, $d_{x^{2}-y^{2}}$), and (*d_xy_*, *d_xz_*) SOC interactions are also negative, which are quite small as compared to that of (*d_yz_*, $d_{z^{2}}$). However, the (*d_yz_*, $d_{x^{2}-y^{2}}$) and (*d_xz_*, *d_yz_*) make small positive contributions. The negative contribution from (*d_yz_*, $d_{z^{2}}$) dominates that of (*d_yz_*, $d_{x^{2}-y^{2}}$ ) and (*d_xz_*, *d_yz_*). The overall contribution is negative, as evidenced in Figure 6. In contrast, by applying the 6% tensile strain, the matrix element differences between (*d_yz_*, $d_{z^{2}}$) and (*d_xz_*, *d_yz_*) change to positive values. At the same time, there is large positive contribution by the (*d_xy_*, $d_{x^{2}-y^{2}}$). The (*d_xy_*, *d_xz_*) and (*d_yz_*, $d_{x^{2}-y^{2}}$) make relatively small negative contributions. Since the magnitude of the matrix element differences between the (*d_xy_*, $d_{x^{2}-y^{2}}$) orbitals are much larger than those of negative ones, the overall MAE for 6% tensile strain gives a relatively strong positive value, i.e., PMA behavior.

**Figure 8:**
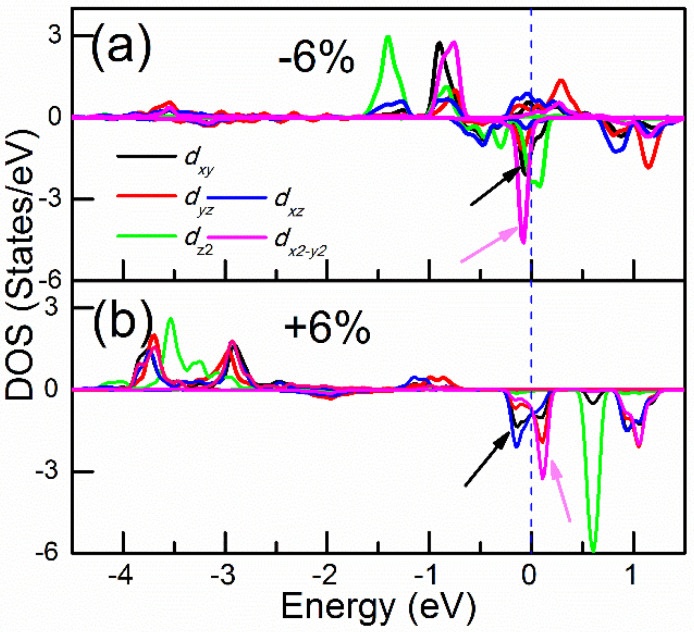
The partial density of states for Fe in −6% (**a**) and +6% (**b**) strains in C-1 stacking configuration. The different orbitals are presented by different colors provided in the figure. The black and pink arrows highlight the changes of the corresponding states. The vertical dashed blue line shows the position of the E_F_.

As shown in Table 2, the SOC interaction between minority (*d_xy_*, $d_{x^{2}-y^{2}}$) orbitals has a large positive contribution. On the other hand, the interfacial hybridization depends on the strength of the orbital overlap and inversely on the energy separation between them. The most important consequence of this hybridization is the formation of the hybridized states in the energies near the E_F_. As shown in Figure 8b, when 6% tensile strain is applied, the minority spin states from the *d_xy_* and *d_yz_* orbitals at −0.15 eV are occupied, whereas the $d_{x^{2}-y^{2}}$, $d_{z^{2}}$ and *d_yz_* orbitals are unoccupied. This is very different from the compressive strain case, as given in Figure 8a, where the minority of spin states of both *d_xy_* and $d_{x^{2}-y^{2}}$ orbitals are occupied near the E_F_ giving negligible contributions to MAE. Furthermore, with tensile strain, the occupied *d_xy_* and the unoccupied $d_{x^{2}-y^{2}}$ orbitals are located very close. Therefore, the $E_{u}^{-}-E_{o}^{-}$ for them is a small value on the denominator. These are the main reasons why the tensile strains favor PMA.

## 4. Conclusions

We have performed DFT calculations to show that valley splitting can occur in monolayer InSe induced by the MPE exerted by ferromagnetic FeI_2_ substrate. The effective Zeeman magnetic field leads to ~48 meV valley splitting, which corresponds to ~800 T magnetic field. The magnetic proximity interaction is mediated by the interfacial orbital hybridizations. As a result, the induced valley splitting shows a strong dependence on the interface distance. The MAE of the system is also studied, which can be tuned by the stacking configurations and the biaxial strains. For the most stable C-1 configuration, the system favors IMA, while it can change to PMA for other stacking configurations. The tensile strains applied on C-1 can also render the system to PMA. This is largely related to the orbital’s hybridization variations at the E_F_, which move the $d_{x^{2}-y^{2}}$ orbital into the conduction band. The large positive matrix element between *d_xy_* and $d_{x^{2}-y^{2}}$ is the major contribution to the PMA for the tensile-strained C-1 system. Our demonstration of valley physics in InSe via MPE broadens its potential applications in spintronic devices.

## Figures and Tables

**Table 1:** Comparison between the different stacking configurations. *d*_0_, *d*_1_ and *d*_2_ are the equilibrium interlayer distance, Fe–Se and Fe–In separation, respectively. *M_F_*_e_, *M_I_*, *M_In_*, and *M_Se_* are the magnetic moments for Fe, I, In and Se in μ_B_, respectively. Δ*E_f_* and Δ*E_v_* are the total energy difference with respect to C-1, and valley splitting at valence band top of InSe, respectively.

	C-1	C-2	C-3	C-4	C-5	C-6
*d*_0_ (Å)	3.227	3.288	3.949	3.950	3.259	3.261
*d*_1_ (Å)	4.808	4.872	5.538	5.5395	4.841	4.845
*d*_2_ (Å)	6.086	6.150	6.8195	6.820	6.119	6.122
*M_Fe_* (μ_B_)	3.37	3.373	3.374	3.375	3.371	3.372
*M_I_* (μ*_B_*)	0.17	0.167	0.168	0.168	0.168	0.168
*M_In_* (μ_B_)	0.004	0.004	0.003	0.003	0.004	0.003
*M_Se_* (μ_B_)	0.005	0.005	0.005	0.005	0.005	0.005
Δ*E_f_* (meV)	0	6	84	84	2	4
Δ*E_v_* (meV)	48.3	48.4	0.2	3.5	51.6	49.9

**Table 2:** The difference of the square of the orbital angular momentum matrix elements between two directions of the magnetization in Equations (5) and (6).

	*o* ^−^					*o^+^*				
*u^-^*	$d_{xy}\;$	$d_{yz}\;$	$d_{z^{2}}\;$	$d_{xz}\;$	$d_{x^{2}-y^{2}}\;$	$d_{xy}\;$	$d_{yz}\;$	$d_{z^{2}}\;$	$d_{xz}\;$	$d_{x^{2}-y^{2}}\;$
$d_{xy}$	0	0	0	−1	4	0	0	0	1	−4
$d_{yz}$	0	0	−3	1	−1	0	0	3	−1	1
$d_{z^{2}}$	0	−3	0	0	0	0	3	0	0	0
$d_{xz}$	−1	1	0	0	0	1	−1	0	0	0
$d_{x^{2}-y^{2}}$	4	−1	0	0	0	−4	1	0	0	0

## Supplementary Materials

The following are available online at https://www.mdpi.com/2079-4991/10/9/1642/s1, Figure S1: The band structures of InSe/FeI_2_ heterostructures with C-2 to C-6 configurations (a–e). The red solid dots indicate the contributions from InSe with their size reflecting the relative weight. Figure S2: Spin density for C-1 (a) and C-3 (b) configurations. The red and blue color represent charge accumulation and depletion, respectively. The isosurface value of 0.0003 e·Å^−3^. The green, pink purple and brown balls represent Se, In, I and Fe atoms, respectively.
