# Supplementary material for: The Magnetic Proximity Effect Induced Large Valley Splitting in 2D InSe/FeI2 Heterostructures
Source: Nanomaterials (Basel). 2020 Aug 21;10(9):1642. doi: 10.3390/nano10091642 (PMC7557779; doi:10.3390/nano10091642)
Supplement: Supplementary file 1 [file nanomaterials-10-01642-s001.pdf]

Supplementary Information

The Magnetic Proximity Effect Induced Large Valley Splitting in 2D InSe/FeI_2 Heterostructures

Yifeng Lin,[1] Changcheng Zhang,[2] Lixiu Guan,[2,*] Zhipeng Sun,[2] and Junguang Tao[1,*]

[1]School of Materials Science and Engineering, Hebei University of Technology, Tianjin 300130, China; Hebut8124@sohu.com (Y.L.)

[2]School of Science, Hebei University of Technology, Tianjin 300401, China; hebut2017@139.com (C.Z.); hebut2016@126.com (Z.S.)

* Correspondence: lixiuguan@hebut.edu.cn (L.G.); jgtao@hebut.edu.cn (J.T.); Tel: +86-15222451579

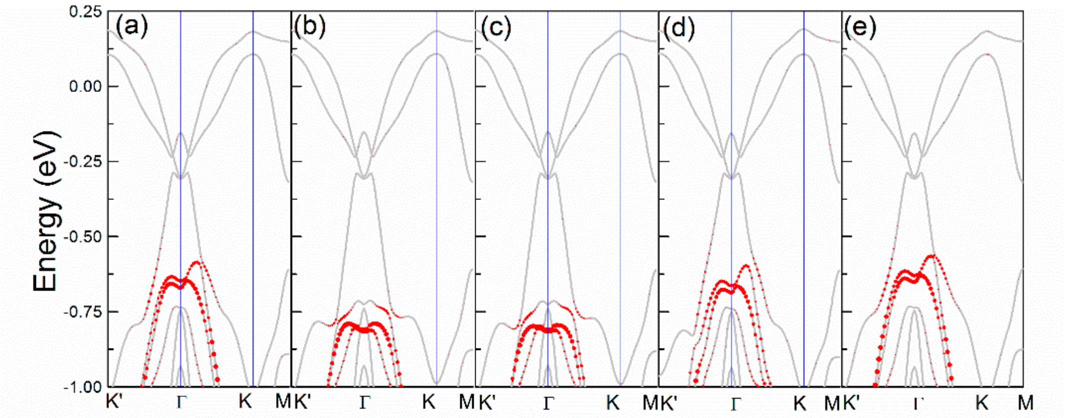

Figure 1. The band structures of InSe/FeI_2 heterostructures with C-2 to C-6 configurations [(a)-(e)]. The red solid dots indicate the contributions from InSe with their size reflecting the relative weight.

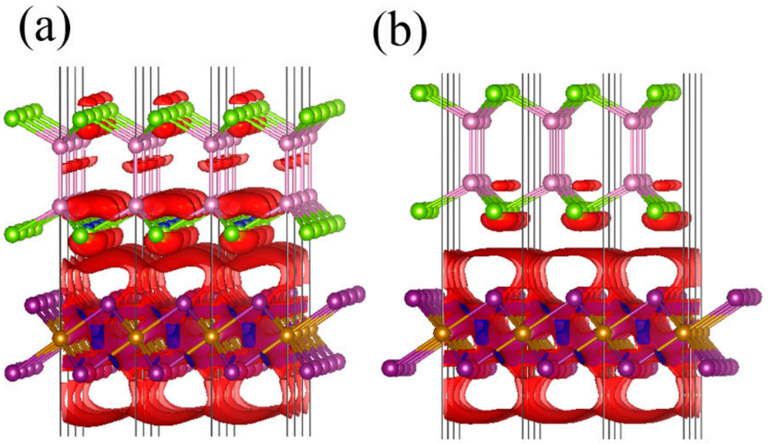

Figure 2. Spin density for C-1 (a) and C-3 (b) configurations. The red and blue color represent charge accumulation and depletion, respectively. The isosurface value of 0.0003 e•Å^{-3}. The green, pink purple and brown balls represent Se, In, I and Fe atoms, respectively.
